# Admission to the ICU overnight: is it really a bad thing?

**DOI:** 10.1186/cc12458

**Published:** 2013-03-19

**Authors:** M Adams, P Dean, K MacDowall, P Stenhouse, A Mackay

**Affiliations:** 1 Victoria Infirmary, Glasgow, UK

## Introduction

Admission to hospital overnight has been shown to increase mortality and decrease hospital length of stay [1]. The objective of this study was to determine whether this relationship is valid in patients admitted to our ICU, and whether length of stay was affected.

## Methods

A retrospective data collection identified 5,827 patients admitted to a five-bed ICU from April 1994 to November 2012. Data regarding patient age, sex, APACHE II score and ICU admission date and time were collected along with the length of stay in the unit and hospital. Definitions of days and night were set to local ICU standards of 9:00 am to 8:00 pm. Patients were then separated into two groups and analysed using Analyse-It software for Excel.

## Results

Crude ICU and hospital mortality rates in patients admitted during the days and overnight were examined. There was no significant difference in unit mortality (day 22.3% vs. night 22.7%, OR = 1.02, 95% CI = 0.91 to 1.16, *P *= 0.718) or hospital mortality (day 30.7% vs. night 29.1%, OR = 0.93, 95% CI = 0.83 to 1.04, *P *= 0.203). The mean unit length of stay showed no difference in patients admitted during daytime compared with those admitted overnight (4.27 days vs. 4.09 days, *P *= 0.162). The mean hospital length of stay was decreased in patients admitted during daystime compared with patients admitted overnight (19.3 days vs. 21.7 days, *P *= 0.004). The average age of patients was less in those admitted out of hours (night 56.5 years vs. days 59.2 years, *P *= <.0001). There was no significant difference in APACHE II scores of patients between the groups (day 19 vs. night 19, *P *= 0.580).

## Conclusion

There is no significant difference between the mortality of patients admitted overnight and patients admitted during the days to our unit. The hospital length of stay is increased in patients who are admitted overnight to intensive care; however, ICU length of stay is not affected. Adjustment for other confounders such as current bed occupancy and staffing ratios during the entire patient stay may help to understand the differences seen in the hospital length of stay.
